# The Impact of IgA and the Microbiota on CNS Disease

**DOI:** 10.3389/fimmu.2021.742173

**Published:** 2021-09-15

**Authors:** Annie Pu, Dennis S. W. Lee, Baweleta Isho, Ikbel Naouar, Jennifer L. Gommerman

**Affiliations:** Department of Immunology, Faculty of Medicine, University of Toronto, Toronto, ON, Canada

**Keywords:** IgA, gut microbiome, ageing, multiple sclerosis, neurodegeneration

## Abstract

Although anatomically distant from the central nervous system (CNS), gut-derived signals can dynamically regulate both peripheral immune cells and CNS-resident glial cells to modulate disease. Recent discoveries of specific microbial taxa and microbial derived metabolites that modulate neuroinflammation and neurodegeneration have provided mechanistic insight into how the gut may modulate the CNS. Furthermore, the participation of the gut in regulation of peripheral and CNS immune activity introduces a potential therapeutic target. This review addresses emerging literature on how the microbiome can affect glia and circulating lymphocytes in preclinical models of human CNS disease. Critically, this review also discusses how the host may in turn influence the microbiome, and how this may impact CNS homeostasis and disease, potentially through the production of IgA.

## Introduction

**Figure 1:**
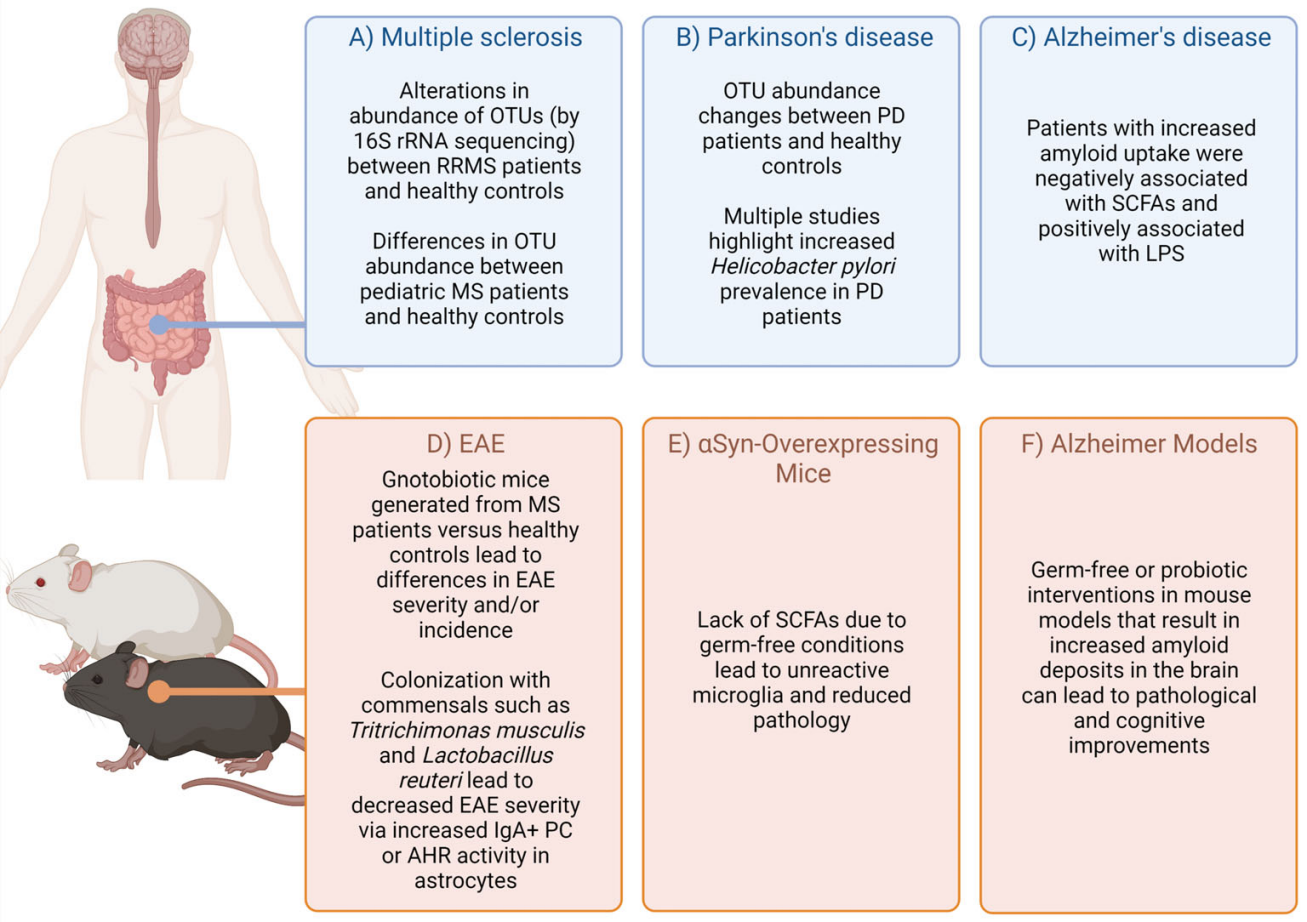
Highlighted evidence for the relationship of microbiota affects in CNS-specific human diseases and animal models. Complex diseases of the CNS are often difficult to query in humans due to scarcity of tissue samples. However, combining evidence from patients, healthy controls **(A–C)** (1–14), as well as animal models **(D–F)** (15–23) can provide some suggestive evidence on how the microbiota may impact disease. Figure made using (BioRender.com).

Complex diseases of the central nervous system (CNS) are caused by a combination of genetic and environmental factors. Human studies and animal models have demonstrated that commensal microbes residing in a host can influence CNS disease (**Figure 1**), adding additional complexity to unraveling the etiology of these diseases. While cheaper and more efficient sequencing technologies has facilitated the human microbiome project (24), we are only at the beginning of identifying disease *vs.* health-promoting microbes, the environmental and genetic factors that promote such communities, and the functional output of these communities.

Two main factors may modify microbiota. First, the microbiota is highly context-dependent and modulated by geographic location and diet (25). Strong evidence suggests diet-based alterations in the microbiome come from extreme diet changes (26) or adoption of new cultural dietary habits (27, 28). There is increasing appreciation for the role of diet-microbiome interactions in CNS diseases (29). Second, internal factors such as host genetics, age, and sex are also important determinants for selecting the gut microbiota (30).

**Figure 2:**
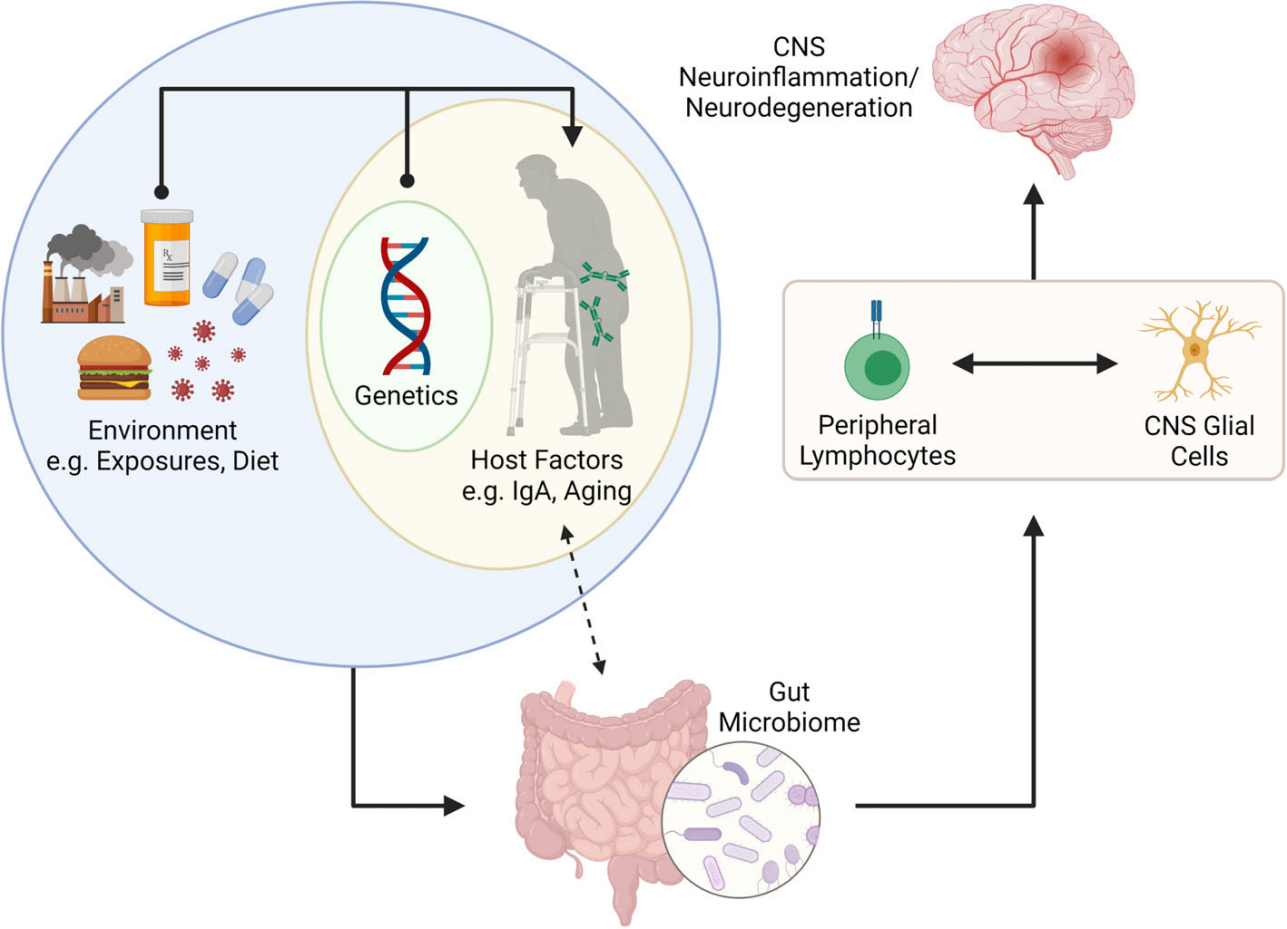
Putative connection between the gut microbiome and CNS neuroinflammation and neurodegeneration. The gut microbiome is shaped by internal (e.g., genetics and other host factors such as mucosal IgA and age) and external factors (e.g., environmental exposures, infections, diet, etc.). Recent literature has suggested that IgA plays a key role in determining the microbes that reside in the gut, but that IgA levels can also be influenced by colonizing microbiota. The gut microbiome is important for programming of peripheral lymphocytes but can also impact the phenotype and function of CNS resident glial cells (*via* metabolites such as SCFAs. The activation (or modulation) of lymphocytes and glial cells can lead to neuroinflammation or neurodegenerative disorders in the CNS. Figure made using (BioRender.com).

Herein we review emerging literature linking host-microbiome interactions to lymphocytes and glial cells in the context of CNS diseases. We describe two key host factors, intestinal IgA and ageing, that have a profound impact on shaping the microbiome. We also provide examples for how these factors impact lymphocytes and glial cells in the context of CNS disease. In summary, we provide a working model for how interactions between host factors (IgA and ageing) contribute towards shaping the microbiome which in turn can influence lymphocyte and glial cell behavior in the context of CNS disease (**Figure 2**).

## Influence of the Microbiota on Lymphocytes in CNS Disease

Correlative data in MS and mouse models demonstrate a bidirectional interaction between the gut and the CNS (15, 16, 31–33); identifying specific contributions of the gut microbiome to CNS disease is imperative for understanding disease pathogenesis. While aberrantly activated lymphocytes are a hallmark of multiple sclerosis (MS), this is less studied in Alzheimer’s and Parkinson’s disease (AD, PD). Thus, this section will focus on the impact of microbiota on lymphocyte function in MS.

### T Lymphocytes

Although alterations in the microbiome have been reported in MS case-control studies (34), testing causal associations between these alterations and disease risk requires animal models. Germ-free (GF) mice lack commensal microbiota and thus present a “blank slate” for exploring the impact of commensal microbes on disease. GF mice fail to develop experimental autoimmune encephalomyelitis (EAE) (35), but when gnotobiotically recolonized or monocolonized with segmented filamentous bacteria (SFB), EAE is rescued. SFB enhances T_H_17 cell induction (36), T cells that are critical in causing pathology in EAE. Regulatory T cells (Tregs) are similarly sensitive to the gut microbiome. Their polarization from naïve T cells can be potentiated by *Bacteroides fragilis* polysaccharide in EAE (17, 35). Interestingly, following transplantation of human MS stool samples into mice (fecal microbiome transplant; FMT), several bacterial species were linked to alterations in T_H_1 and Treg differentiation, and consequently EAE phenotype (37, 38).

### B Lymphocytes

B cells produce antibodies, present antigen to T cells, and secrete cytokines. When antigen binds to a B cell receptor, these antigen-specific B cells are activated and undergo somatic hypermutation and affinity maturation in germinal centers (GC) (39), generating high-affinity antigen-specific receptors. GC B cells can also class-switch to generate different antibody isotypes with specialized effector functions (IgA, IgE, IgG). Mature B cells can also differentiate into memory B cells or antibody-secreting cells (ASCs) (40). ASCs comprise both proliferative plasmablasts (PBs) and terminally differentiated plasma cells (PCs) (39, 41). Alterations in microbial abundance have been correlated with changes in regulatory B cell (Breg) induction (18, 42). Antibiotic treatment enhanced frequencies of IL-10-producing Bregs at baseline and in EAE (42). In addition, a role for gut-derived commensal-reactive IgA^+^ ASCs in attenuating EAE and possibly also MS has been shown (18, 19), described below.

## Influence of the Microbiota on Glial Cells in CNS Disease

Glial cells have been intensively studied in each MS, AD, and PD, and a role for the microbiome in modulating glial cell phenotype and function in these diseases is emerging.

### Microglia

Microglia are CNS-resident macrophages serving key homeostatic and immune functions in the developing and adult CNS (1). Maternal microbiota can influence microglial maturation and function during both fetal development and in adulthood, as demonstrated in GF and antibiotic-treated mice (20, 21). Given the lack of evidence for a CNS microbiome, presumably microbiota-derived metabolites, such as short-chain fatty acids (SCFAs), directly or indirectly influence microglial phenotype (43). Microglia in GF mice are not fully mature, and interestingly, colonization with altered Schaedler’s flora failed to rescue microglial defects whereas SCFA supplementation reversed the abnormal phenotype (21), indicating that the presence of SCFA-producing bacteria or a diverse microbiota are necessary for maturation.

In models of MS, antibiotic treatment prior to lysolecithin (LPC)-induced demyelination decreases microglia activation, indicated by the reduction in intralesional P2ry12^lo^Clec7a^+^ inflammatory cells as opposed to P2ry12^+^Clec7a^-^ homeostatic microglia. Microglial activation was also attenuated in GF mice given the demyelinating toxin cuprizone (44). Surprisingly, supplementing aged mice with probiotic VSL#3 enhanced serum and fecal SCFAs, but had limited effect post-LPC administration (44). Indoxyl-3-sulfate (I3S), a product of microbial tryptophan metabolism, activates aryl hydrocarbon receptor (AHR) on microglia, augmenting TGFα production to limit astrocytic inflammation in EAE (45). Feeding whole milk promotes AHR ligand and SCFA production and ameliorates EAE in marmosets, although the effect is not attributed solely to microglia, but overall modulation of inflammation (46).

In α-synuclein-overexpressing (ASO) mice that model PD, the presence of a gut microbiota promotes the aggregation of α-synuclein in the caudoputamen and substantia nigra, resulting in microglial activation and motor dysfunction (47). GF ASO mice show significantly decreased levels of aggregated α-synuclein and are protected from development of motor deficits. Re-colonization of GF mice with SPF microbiota reversed this rescue effect. Surprisingly, despite SCFAs being generally thought to be anti-inflammatory, this study inculpates SCFAs in promoting aggregated α-synuclein. SCFA supplementation to GF or antibiotic-treated ASO mice increased microglial activation and is sufficient to promote motor impairment. In addition, abundance of several SCFA-producing enzymes is increased in humanized mice that have received an FMT from Parkinson’s disease donors.

Like other neurodegenerative diseases, an unhealthy microbiome is increasingly appreciated as a risk factor for AD. Similar to PD, circulating levels of the SCFAs acetate and valerate were positively correlated with Aβ load in the brains of AD patients (48). Mice treated with probiotic bacteria exhibited ameliorated AD-like cognitive decline and decreased Aβ aggregation (49). GF 5x Familial Alzheimer’s disease (5xFAD) mice show a decreased Aβ load in the hippocampus compared to conventional 5xFAD mice, attributed to the uptake of Aβ debris by microglia. A greater number of Iba1^+^ cells were found in the hippocampus closely associated with Aβ plaques in GF 5xFAD mice, and a higher percentage of these plaque-associated Iba1^+^ cells were positive for methoxy-X-O4, an indicator of Aβ uptake (50).

### Astrocytes

Astrocytes play diverse roles in the homeostatic brain that include providing trophic support to other CNS cells, regulation of synaptic activity, and controlling the blood-brain barrier (51–54). Astrogliosis is also a feature of MS. Unsurprisingly, astrocytes play important roles in modulating neuroinflammation, as they can produce inflammatory cytokines and a host of chemokines that promote chemotaxis of other immune cells. In EAE, astrocytic inflammation is shown to be directly attenuated by I3S activation of AHR (45). Gut microbiota depletion by antibiotic treatment decreases levels of I3S and worsens EAE disease (55). AHR-deficient astrocytes increase expression of several pro-inflammatory chemokines and cytokines. Importantly, IFNβ, a therapeutic used in some MS patients, works to limit CNS inflammation through this mechanism, as the anti-inflammatory effect of IFNβ is lost in AHR-deficient astrocytes (55).

### Oligodendrocytes

Oligodendrocytes were previously thought to be quiescent, myelin-producing cells. However, increasing evidence shows that oligodendrocytes actively communicate with and provide metabolic support to neurons (56). Mature oligodendrocytes may also participate in remyelination and are active players during neurodegeneration (57). However, little is known about interactions between oligodendrocyte lineage cells and the gut microbiome. While treatment of mice with the probiotic VSL#3 enhanced SCFA concentrations in feces and serum, there was no effect on remyelination *in vivo* following LPC-induced demyelination (44). Conversely, a separate study found that in *ex vivo* organotypic cerebellar slice cultures demyelinated by lysolecithin, the addition of the SCFA member butyrate enhanced both OPC numbers and mature oligodendrocyte numbers, indicating a positive effect on remyelination (58). In summary, the gut microbiota exerts effects not only on peripheral immune compartments, but also act on glial cells, with potential impacts on CNS disease processes.

## Host Factors That Influence the Intestinal Microbiome – A Focus on IgA and Ageing

Many external factors influence the gut microbiome such as diet and pathogen exposure. In this section, we review host factors that shape the microbiome, focusing on IgA and ageing.

### Host Control of the Microbiota Through IgA

Mature B cells primed in Peyer’s patches can differentiate into IgA-producing PCs and home to the intestinal lamina propria (41, 59, 60). The IgA produced by GALT PCs is typically dimeric, joined through the J-chain (41, 61). Secretory IgA is generated when dimeric IgA binds the polymeric-Ig-receptor (pIgR) on the basolateral surface of the intestinal epithelium, translocates through the epithelial cells, and is released into the lumen with the secretory component of the pIgR.

In mice, IgA both contributes to host control of microbiota and is responsive to gut microbiota changes. Mice monocolonized with *Bacteroides thetaiotamicron* harbor a reduced IgA repertoire restricted to a single clone against the capsular polysaccharide of the bacterium (62). Oral administration of *Lactobacillus casei* to mice resulted in an increase in IgA^+^ cells in the small intestinal lamina propria (SILP) (63). Exposure of conventional mice to commensal Proteobacteria also resulted in increased serum IgA levels and induction of IgA^+^ PC in the bone marrow (64). In contrast, some microbial communities can diminish IgA levels in the lumen due to their ability to degrade the secretory component (65). Even strain level differences in the microbiome can dictate IgA levels in the host (66). Conversely, the host IgA response can dictate the composition of the microbiome. Activation-induced cytidine deaminase-deficient mice (which fail to produce competent IgA), exhibit an expansion of SFB in the small intestine which leads to isolated lymphoid follicle (ILF) hyperplasia and GC enlargement in secondary lymphoid tissues (67, 68). Restoration of IgA by heterogenetic parabiosis with wild-type mice reduced SFB populations, ILF protrusion, and spleen and lymph node size.

In humans, modest alterations in fecal microbiota composition are observed in subjects with selective IgA-deficiency (SIgAd) (69, 70). Compensatory sIgM in SIgAd subjects has a distinct bacterial binding pattern: an unclassified *Enterobacteriaceae* taxon heavily coated by IgA in healthy controls and by IgM in selective IgA-deficient subjects, was significantly more abundant in SIgAd subjects, demonstrating that IgA coating specifically restricts expansion of this taxon, and the same effect is not achievable by IgM.

Overall, these data indicate that in both mice and humans, a bi-directional relationship exists between host IgA and gut microbiota.

### Impact of Ageing on the Microbiome

Growing evidence suggests that the gut microbiota has a critical impact on the ageing process and is a possible determinant of healthy ageing (71–73). Cross-sectional studies have examined alterations in the microbiota composition across the human lifespan (74, 75), demonstrating that taxonomical composition of gut microbiota appears to follow stepwise progression through life. Taxonomic shifts in the microbiota and decrease in microbial richness and diversity are observed in frail older individuals and associated with worse health outcomes compared to younger individuals (76–80). Relative abundance of Ruminococcaceae, Lachnospiraceae, and Bacteroidaceae families decrease with age, whereas an enrichment and/or higher prevalence of health-associated genera such as *Akkermansia*, *Bifidobacterium*, and Christensenellaceae, are maintained in longevity and extreme longevity (81). Indeed, centenarians tend to exhibit indicators of good health, and greater gut microbiota complexity (74). The relative abundance of pathobionts decreases and beneficial commensals, such as *Akkermansia*, are retained (82). Studies that stratify between elderly and centenarian status identify changes in taxa associated with extreme longevity including *Odoribacter*, *Butyricimonas*, *Desulfovibrio*, *Bilophila*, *Oscillospira*, and *Akkermansia* genera, and the Christensenellaceae and Barnesiellaceae families (75, 81).

Similar taxonomic and functional patterns that correlate with age and frailty in the mouse microbiome have been identified (83). In aged mice, the ratio of Firmicutes to Bacteroidetes increased ∼9‐fold compared to young mice, indicating dysbiosis, although this work was performed in commercially purchased mice rather than mice derived from the same dam (84). Introducing aged microbiota to young mice increased mortality following ischemic stroke, decreased behavioral outcomes, and increased cytokine levels. Conversely, altering the microbiota in aged mice to resemble that of young mice increased stroke survival and improved recovery (84). Changes in the gut microbiota in aged mice were also associated with increased gut permeability and elevations of peripheral inflammation (85, 86). Taken together, except for healthy centenarians who resist frailty, ageing is associated with an unhealthy microbiome.

## Influence of Ageing and IgA on CNS Disease *via* the Microbiome

Multiple internal host factors impact the microbiome, including IgA and ageing. Here we speculate on how these two host factors may impact brain health and the trajectory of brain disease *via* the microbiome.

### IgA, the Microbiome, and CNS Disease

Although IgA^+^ ASCs can home to the dura mater during homeostasis (87), clonally expanded IgA are absent in steady state CNS and only appear during inflammation (88–91). During EAE, a significant reduction in IgA^+^ ASCs is apparent in the SILP. Additionally, adoptively transferred gut-derived IgA^+^ ASCs were found in the CNS were reactive to mouse-derived gut bacteria and were shown to alleviate neuroinflammation by producing IL-10 at chronic stages of EAE. Over-abundance of IgA^+^ ASCs was able to reduce GM-CSF production by T cells, an important cytokine that promotes neuroinflammation (18). *Tritrichomonas musculis* (*T.mu*) is a rodent commensal that promotes IgA production (92). EAE incidence and severity, as well as spinal cord inflammation and demyelination, are reduced in *T.mu^+^* mice (18). *T.mu^+^* mice also exhibited elevated serum and fecal IgA levels and increased frequencies of IgA^+^ ASCs in the gut, bone marrow, and brain.

While the above highlights key findings from animal models, there is also early evidence suggesting the importance of the microbiota-driven IgA response in human disease. Bacteria identified by IgA-seq were differentially expressed in MS patients *versus* healthy controls (19). Stratified by disease activity, MS patients in relapse exhibited decreased percentages of IgA-bound gut bacteria in fecal samples compared to remitting patients, with corresponding elevation in CSF IgA. CNS-infiltrating IgA^+^ B cells show specificity for gut microbial antigens, indicating the migration of IgA-producing cells from the gut during relapse. IgA is also elevated in cerebrospinal fluid of MS patients. Importantly, commensal-specific IgA^+^ ASCs have been observed in inflammatory lesions of MS patients (19). This phenomenon may not be IgA-exclusive, however, as IgG in MS patient CSF has been found to be reactive against MS-associated gut bacterial lysate (93). The implications of these bacteria-reactive IgG in disease have yet to be fully elucidated.

Lastly, while IgA^+^ ASC have been now described in the inflamed EAE and MS CNS, it is now appreciated that these cells play an important role in homeostasis. Specifically, intestinal commensal specific IgA^+^ ASC have been detected in the leptomeninges of healthy mice and humans but are absent in GF mice (87). These cells likely maintain barrier integrity near the dural sinuses; however, it is possible they may also contribute to quiescence within the CNS.

In summary, in addition to its well appreciated role in shaping the microbiome, IgA-producing ASC likewise play important roles in the healthy and MS/EAE CNS. The role for these cells in PD and AD is not yet understood.

### Ageing, the Microbiome, and CNS Disease

Ageing is the predominant risk factor for neurodegenerative diseases (94), yet in spite of the known role ageing has on the microbiome, the connection between ageing, the microbiome and CNS disease has barely been explored.

It is well established that microglia are affected during ageing. Ageing results in decreased number, uneven distribution, lower motility, and fewer ramifications, as well as impairment in phagocytosis and injury responses (2, 95–98). Senescent microglia increase pro-inflammatory cytokine production (3). An altered microglia morphology and reduced arborization have been observed in the human brain during ageing and age-related diseases such as AD (95). This dystrophic morphology is associated with impaired spatial learning (3).

Age-related changes in the gut microbiome may have a direct impact on microglial function within the CNS. In fact, reduced complexity of microbiota, a feature of ageing, leads to defects in microglia maturation and function (21). Recent work demonstrated that FMT from aged donor mice into young recipients impairs spatial learning and memory in young recipients (4). Conversely, FMT from young donor mice into aged recipients can rejuvenate age-associated CNS metabolic, transcriptomic, and behavioral changes (5). Aged into young FMT induced an altered expression of proteins involved in synaptic plasticity and neurotransmission in the hippocampus, an area of the CNS known to be affected by the ageing process. A strong reduction of bacteria associated with SCFA production (Lachnospiraceae, *Faecalibaculum*, and Ruminococcaceae) and disorders of the CNS (Prevotellaceae and Ruminococcaceae) was also reported (4). Interestingly, microglia of the hippocampus acquired an ageing-like phenotype following FMT. Of therapeutic relevance, this age-associated phenotype can be reversed by re-introducing live and complex microbiota or microbial metabolites, such as SCFAs (6).

The gut microbiota similarly affects astrocytes in both ageing and age-associated neurodegenerative diseases (7). Ageing can alter the normal function of astrocytes which reduces their ability to properly maintain a healthy CNS environment (8). Astrocyte transcriptomes from multiple mouse brain regions have revealed that ageing upregulates genes that eliminate synapses and induces a reactive astrocyte gene signature (9). Therefore, aged astrocytes may promote synapse elimination and neuronal damage, contributing to ageing-associated cognitive decline. Morphological changes in astrocytes have also been documented in the aged CNS (10–12). Aged astrocytes increase cytokine production, notably CXCL10 (13) that attracts peripheral immune cells and promotes T cell adhesion to endothelial cells (14). CXCR3, which is the CXCL10 receptor, is expressed in microglia, suggesting that astrocytes and microglia communicate during ageing (22, 45).

## Conclusions

Chronic, complex diseases of the CNS develop over years. Animal studies conducted under controlled conditions in short periods miss two large confounders in these diseases – time (ageing) and the microbiota-IgA axis, with age-associated microbiota alterations further complicating this relationship. These are important considerations for animal modelling, given the considerable variability in microbiota composition and gut luminal IgA levels between vivaria (65). In summary, we propose that host factors such as age and intestinal IgA are key determinants in how the microbiome impacts lymphocyte and glial cell phenotype/function in the context of MS, AD and PD (**Figure 2**).

## Author Contributions

AP, DL, BI, and IN all contributed to the writing of this manuscript. AP, DL, and JG contributed to the editing of the text and generation of all figures. All authors contributed to the article and approved the submitted version.

## Conflict of Interest

The authors declare that the research was conducted in the absence of any commercial or financial relationships that could be construed as a potential conflict of interest.

## Publisher’s Note

All claims expressed in this article are solely those of the authors and do not necessarily represent those of their affiliated organizations, or those of the publisher, the editors and the reviewers. Any product that may be evaluated in this article, or claim that may be made by its manufacturer, is not guaranteed or endorsed by the publisher.
